# The Effect of Antagonist Muscle Sensory Input on Force Regulation

**DOI:** 10.1371/journal.pone.0133561

**Published:** 2015-07-17

**Authors:** Tanya Onushko, Brian D. Schmit, Allison Hyngstrom

**Affiliations:** 1 Department of Biomedical Engineering, Marquette University, Milwaukee, Wisconsin, United States of America; 2 Department of Physical Therapy, Marquette University, Milwaukee, Wisconsin, United States of America; The University of Queensland, AUSTRALIA

## Abstract

The purpose of this study was to understand how stretch-related sensory feedback from an antagonist muscle affects agonist muscle output at different contraction levels in healthy adults. Ten young (25.3 ± 2.4 years), healthy subjects performed constant isometric knee flexion contractions (agonist) at 6 torque levels: 5%, 10%, 15%, 20%, 30%, and 40% of their maximal voluntary contraction. For half of the trials, subjects received patellar tendon taps (antagonist sensory feedback) during the contraction. We compared error in targeted knee flexion torque and hamstring muscle activity, with and without patellar tendon tapping, across the 6 torque levels. At lower torque levels (5%, 10%, and 15%), subjects produced greater knee torque error following tendon tapping compared with the same torque levels without tendon tapping. In contrast, we did not find any difference in torque output at higher target levels (20%, 30%, and 40%) between trials with and without tendon tapping. We also observed a load-dependent increase in the magnitude of agonist muscle activity after tendon taps, with no associated load-dependent increase in agonist and antagonist co-activation, or reflex inhibition from the antagonist tapping. The findings suggest that at relatively low muscle activity there is a deficiency in the ability to correct motor output after sensory disturbances, and cortical centers (versus sub-cortical) are likely involved.

## Introduction

The integration of sensory feedback with descending motor commands is important for controlling force output during voluntary movements. Muscle afferent pathways and descending pathways from supraspinal centers regulate the magnitude and timing of motoneuronal activity [1,2]. While it is generally known how muscle afferent feedback affects force regulation within the agonist muscle, less is known about sensory integration between agonist and antagonist muscle groups in humans. Understanding the effects of sensory integration from muscle afferent pathways between agonist and antagonist muscles during sub-maximal contractions is important not only from a basic motor control standpoint, but also because it has clinical implications. Alterations in the excitability of these pathways have been noted in patient populations, such as stroke, and can be detrimental to force regulation and movement [3,4].

In recent years, several research groups have quantified the regulation of sub-maximal leg forces and demonstrated differences between patient populations and healthy controls [5–8]. Sub-maximal force regulation has been used as a probe to understand volitional control strategies and expose sub-clinical impairments in force regulation. Typical measurements include the ability to achieve a target force (error) and the ability to maintain a given force (steadiness). Although force regulation can be task and muscle specific, larger muscle groups of the leg tend to have worse force regulation at lower relative force levels (< ~20% of maximal voluntary contractions) as compared to higher force levels [8,9]. This manifests as decreased steadiness or increased error at lower force levels, and correlates with clinical measures of function [7]. The load-dependent effect on sub-maximal force regulation can be attributed to motoneuron:muscle fiber innervation ratios (lower resolution of control) [8] and, in some cases, poor temporal regulation of agonist:antagonist pairs [9–11].

In humans, the interaction of sensory feedback and volitional regulation of force has largely been examined by manipulating sensory feedback within the agonist muscle. During isometric contractions, vibrating the agonist muscle excites Group Ia pathways and subsequently, creates force errors in motor output [12]. Specifically, Jones and Hunter [12] demonstrated a load-dependent effect of agonist sensory feedback during constant isometric contractions of the elbow flexors. Subjects produced higher errors at force levels <60% of their maximal voluntary contraction (MVC), while at force levels >60% MVC subjects generated lower errors in force [12]. Similarly, a history of maximally contracting a muscle has been shown to decrease responsiveness in golgi tendon organs of the agonist and result in larger errors in subsequent force output [13]. Less is known about sensory integration and force regulation between agonists:antagonists.

There is some evidence that the integration of sensory information from muscle afferents, particularly Group Ia pathways, and descending inputs exists in reciprocal muscle pairs. Aimonetti et al. [14] demonstrated that stimulation of mixed nerves within the antagonist muscle during an isometric contraction affects the firing probability of different agonist motor unit types in the human arm. The stimulation increased the probability of activation of faster firing motor units (Type II), which are typically recruited at higher forces, while simultaneously decreasing firing probability in slower firing motor units (Type I) [14]. These results suggest a control strategy that depends on the magnitude of the contraction. In another study, Kudina [15] examined patellar tendon tapping on motor unit activity during weak contraction of the biceps femoris muscle. Although the methodology did not include measures of force regulation, the authors showed that new motor units were recruited in the biceps femoris muscle. The authors conclude that the history of patellar tap reflex response increased the excitability of the agonist muscle motor units in a subsequent contraction. An important remaining question is whether sensory integration of Group Ia inputs and volitional regulation of force may also exist between agonist-antagonist muscle pairs and what the effect of load (and hence degree of agonist muscle activation) may be.

For this study, we examined how sensory feedback from Ia pathways in knee extensors affects knee flexor activity during various isometric contraction levels. Our purpose was to understand how stretch-related sensory feedback from the antagonist muscle affects agonist motor output at different contraction levels in healthy adults. To test this, subjects performed isometric knee flexion contractions (agonist) at different torque levels with and without patellar tendon taps (antagonist sensory feedback). We hypothesized that patellar tendon taps would exacerbate error and variability at lower load levels (< 20% MVC) because motor output error and variability is greater at lower contraction levels and sensory feedback affects force regulation.

## Methods

### Participants

Ten healthy men (mean age ± SD: 25.3 ± 2.4 years) were recruited for this study. None of the subjects reported any current or past neurological pathology. All research was approved by the Marquette University's Institutional Review Board (IRB, HR-1196). All clinical investigation were conducted according to the principles expressed in the Declaration of Helsinki. Written, informed consent was obtained from the participants.

### Experimental Setup and Approach

**Fig 1:**
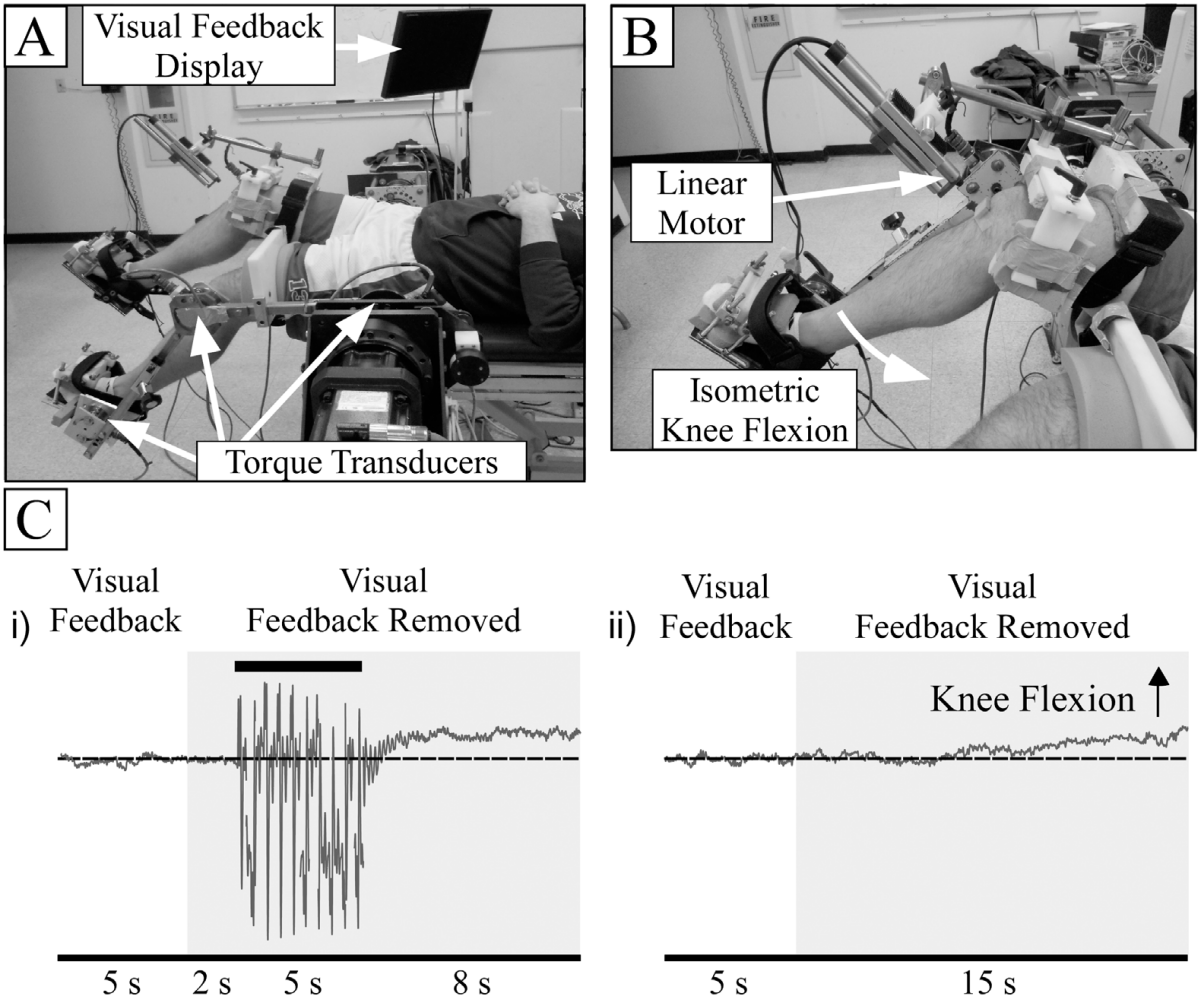
Experimental Setup. (A) Robotic apparatus used to measure knee torque during isometric contractions. Subjects were given visual feedback on a computer monitor during the initial 5 seconds of the trial to ensure they reached the correct contraction level. (B) A linear motor provided controlled tendon taps to the right patellar tendon. Ten tendon taps (2 Hz and 25 ms duration) were applied 2 seconds after visual feedback was removed. (C) Experimental time line for isometric knee flexion contraction with (i) and without (ii) tendon taps. After 5 seconds, visual feedback of knee flexion torque was removed for the remainder of the trial.

Subjects lay supine on a height-adjustable table with their legs secured into a robotic test apparatus to acquire knee torque measurements (Fig 1A) [16]. The robotic apparatus consisted of custom-built leg braces attached to two servomotor systems (Kollmorgen, Northhampton, MA). The participant’s legs were secured within the leg braces using a strap around the thigh, a strap around the ankle, and a clamp over the dorsum of the foot that secured the foot to a footplate at the end of the brace. The right leg was used as the test leg for all subjects and all subjects self-reported right-side dominance.

A custom-designed motorized reflex hammer was attached to the right leg brace and was used to deliver controlled patellar tendon perturbations (Fig 1B). Tendon taps activate Ia afferents [17] and the tendon tap or jerk response to tendon taps is the gold standard in the clinic for measuring stretch reflex responses for neurologic physical examinations [18,19]. The motorized hammer consisted of a LinMot P series linear motor (P01-23x160/70x70) powered by a LinMot E1010 amplifier (LinMot Inc., Delavan, WI). A small rubber tip (12 mm diameter) was screwed into the end of the motor shaft. After palpating the knee to locate the patellar tendon, the rubber tip was placed 5 cm from the tendon and aligned perpendicular (~90°) with respect to the tibia. A rubber pad (2.8 x 3.1 cm) was taped to the subject’s patellar tendon to distribute the force of the tendon perturbation. A linear variable differential transformer (LVDT; Accusens Series 2000 DC-EC, Measurement Specialists Inc., Hampton, VA), attached to the linear motor shaft, was used to measure the motor’s displacement during trials with tendon tapping. Custom-written LabVIEW programs controlled the linear motor’s velocity.

#### Torque measurements

Knee torque was measured from a torque transducer (S. Himmelstein and Company, Hoffman Estates, IL) integrated within the leg brace. The torque transducer axis was aligned with the approximate anatomical center of knee rotation. Torque signals were sampled at 2 kHz using a data acquisition card (National Instruments Corp., Austin, TX) and low-pass filtered (500 Hz analog filter) prior to acquisition.

#### EMG measurements

Surface electromyograms (EMGs) were recorded from five muscles of the right leg: vastus medialis (VM), vastus lateralis (VL), rectus femoris (RF), medial hamstrings (MH), and lateral hamstrings (LH). Disposable, pre-gelled electrodes (Vermed Medical Inc., Bellows Falls, VT) were placed over the muscle belly in a bipolar arrangement. The skin was cleaned with alcohol prior to electrode placement. EMG signals were pre-amplified (x 1000) and filtered (band-pass 10–1000 Hz) (Bortec Medical AMT-16; Calgary, Alberta, Canada) before digitization. EMG signals were sampled at 2 kHz using a data acquisition card (National Instruments Corp., Austin, TX) and a PC.

#### Maximal voluntary contractions

Before beginning the experimental testing, subjects performed isometric maximal voluntary contractions (MVCs) of the right knee flexors. Visual feedback displayed on the computer monitor cued subjects when to contract and relax for each MVC trial. Subjects held the contraction for 5 seconds and were verbally encouraged throughout each MVC trial. Trials were repeated (up to 5 trials) until the maximal knee flexion torque from 2 of the 3 most recent trials was less than 5% of each other. The MVC was quantified as the average torque maintained for 2 seconds of the trial with the highest torque. During the MVC trials, the right leg (test leg) was positioned at 20° hip flexion and the left leg was positioned at 0° hip flexion.

#### Constant isometric task

Subjects performed submaximal isometric knee flexion contractions with the right leg. They were asked to match 6 constant target torque levels (5, 10, 15, 20, 30 and 40% MVC) and maintain the contraction for 20 s. The target was displayed on a computer monitor as a horizontal line and subjects were given visual feedback of their torque to ensure they reached the correct level. Once subjects reached and maintained the contraction level for 2 seconds, visual feedback was given for 5 additional seconds and then removed for the duration of the trial (15 s; Fig 1C). For each target level, subjects performed 3 trials with patellar tendon tapping (TAP) and 3 trials without patellar tendon tapping (NTAP) while maintaining the contraction. During the TAP trials, 10 tendon taps (2 Hz and 25 ms duration) were applied 2 seconds after visual feedback was removed (Fig 1C). An additional trial with tendon tapping was performed while subjects were at rest (0% MVC). Each target level was presented in random, block order (13 trials per block x 3 blocks) for a total of 39 trials. Subjects were given 30–60 seconds rest between trials to limit the effects of fatigue.

### Data Analysis

Data were analyzed using custom-written Matlab programs (Math Works Inc., Natick, MA, USA). Prior to further analyses, the torque recordings were low-pass filtered (20 Hz) and the EMG recordings were band-stop filtered (60 Hz) and band-pass filtered (10–300 Hz) using 2^nd^ order Butterworth filters (bi-directional). Torque and EMG signals were then divided into two 5-second segments—the first 5 seconds (PRE) and the last 5 seconds (POST) of the constant portion of the contraction.

#### Torque measurements

**Fig 2:**
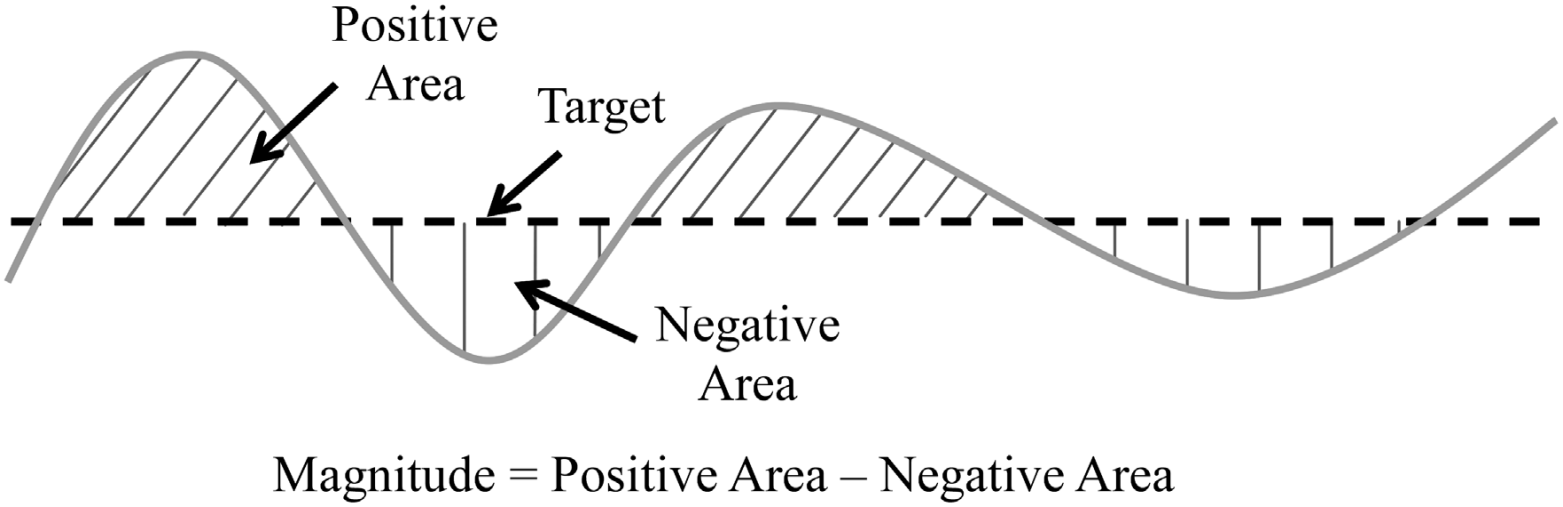
Torque magnitude example calculation. Torque magnitude was calculated as the difference between the positive area and negative areas of the knee flexion torque relative to the target.

We quantified the following for the POST segment of torque data: variability, error, and magnitude. Variability was quantified as the coefficient of variation (CV, standard deviation / mean) of the torque signal. Prior to calculating the CV, the torque signal was first linearly detrended to minimize any drift of the signal. Detrending the data is the accepted protocol for determining the CV of force because any drift occurring in the torque recording, especially during the no visual feedback condition, could influence the relative magnitude of the variability [8, 20–22]. Errors in tracking accuracy were quantified using the root mean square error (RMSE) with respect to the target torque level. Magnitude of torque was quantified to determine the amount of torque subjects produced relative to the target. We quantified the magnitude of the torque by calculating the area of the torque signal above and below the target, and then taking the difference between the area greater than and the area less than the target level (Fig 2). For the PRE segment of the torque data, we quantified the RMSE with respect to the target level.

#### Neural activation of muscles

We examined muscle activation using three measures from the EMGs: 1) EMG magnitude of the hamstrings during contraction, 2) peak-to-peak amplitude of the quadriceps EMG in response to patellar tendon taps, and 3) co-activation ratio for the MH and quadriceps EMG. The magnitude of activation of the MH EMG was based on the root mean square (RMS) values, normalized to the respective EMG MVC. We then calculated the percent change (Δ%) of the MH and LH EMG magnitude from POST to PRE (100*[POST-PRE]/PRE). Next, we identified the peak-to-peak (P-P) reflex amplitude from the RF, VL, and VM EMG during trials with tendon tapping. The peak to peak amplitude (the difference in volts between the positive and negative peak of the EMG signal) was used to measure the reflex response because the recorded reflex EMG signal is tri-phasic and the peak to peak response accounts for the maximum of both the positive and negative phases [18, 23–24]. The tendon tap EMG response was differentiated from background EMG based on the latency from the tap (~20 ms) and its standard tri-phasic shape. The P-P reflex amplitude at each target level was normalized to its respective P-P EMG reflex response during the 0% MVC trial. We averaged the P-P RF, VL and VM reflex responses within each target level. We did this to minimize the variability in the quadriceps EMG [25, 26]. Last, we calculated a co-activation ratio of quadriceps and hamstring activity. This was computed as the sum of the average quadriceps EMG (RF, VM and VL) RMS amplitude divided by the MH EMG [27, 28]. We did not use the LH EMG in our analyses because LH was not consistently recruited between subjects and within subjects. We used the MH EMG for analysis because it was consistently activated. The co-activation ratio was calculated only during the POST segment for each target level.

#### Frequency analysis

A spectral analysis was performed on the torque, and on the MH EMG PRE and POST segments to examine oscillations in motor output. Comparisons were made between TAP and NTAP conditions. The spectral data were obtained using Welch’s average periodogram method with a non-overlapping Hanning window (Matlab). The length of each segment was 5 seconds and the window size was 10,000 points, which resulted in a frequency resolution of 0.2 Hz. The torque segments were linearly detrended prior to the spectral analysis. For statistical comparisons, the frequency of the torque signal was divided into 0.1–1, 1–3, 3–7 and 7–10 Hz frequency bands [29]. We quantified the percent peak power within each of the bands. The percent peak power was calculated as the peak power within each frequency band relative to the sum of peak powers within all bands (0.1–10 Hz). The EMG segments were analyzed similarly. The EMG segments were first detrended prior to spectral analysis. Then the spectrum was divided in to 3 frequency bands (13–30, 30–60 and 60–100 Hz) because these muscle discharge frequencies have been previously associated with specific cortical drives [30]. The EMG power in each band was normalized by the total peak power across all frequency bands (13–100 Hz). The percent change (Δ%) from POST to PRE (100*((POST-PRE)/PRE) was calculated for the torque and MH data.

### Statistical Analysis

A two-way ANOVA (6 target levels x 2 tendon tap conditions) with repeated measures on both factors was used to compare the torque variability, error, and magnitude measures of the hamstrings EMG, and co-activation ratio. A three-way ANOVA (4 frequency bands x 2 tendon tap conditions x 6 target levels) with repeated measures on all factors was used to compare the relative peak power in the torque power spectrum. Similarly, a linear mixed-model ANOVA (3 frequency bands x 2 tendon tap conditions x 6 target levels x subject (random factor)) with repeated measures on frequency bands, tendon tap condition and target levels was used to compare the normalized EMG power in the MH EMG signals. The P-P reflex EMG resulted in non-normal distributions. Thus, we used Friedman’s ANOVA to determine whether the normalized EMG P-P reflex amplitude was statistically different among the 6 target levels.

Statistical analyses were performed using IBM SPSS Statistics 21.0 statistical package (IBM Corp., Armonk, NY, USA). Appropriate post-hoc tests were performed on significant interactions from the ANOVA models. Multiple pairwise comparisons were corrected using Bonferroni corrections. The alpha level for all statistical tests was 0.05. Only significant main effects and interactions are presented, unless otherwise stated.

## Results

### Effects of antagonist Ia sensory feedback on motor output

**Fig 3:**
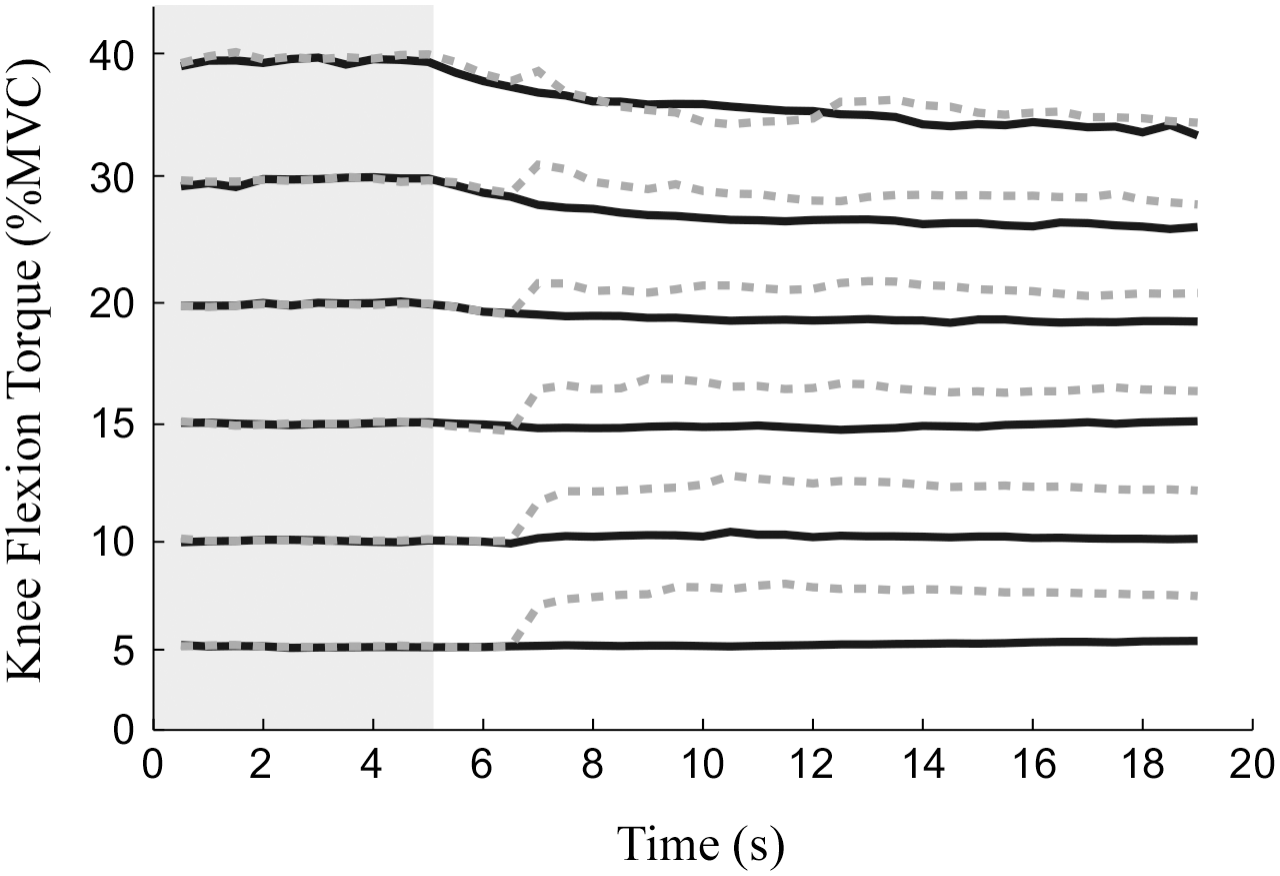
Average knee flexion torque. TAP trials (light, dotted lines) and NTAP trials (dark, solid lines) are illustrated for each target torque level as a percent of the MVC. Shaded area represents the time visual feedback was given to the subjects.

**Fig 4:**
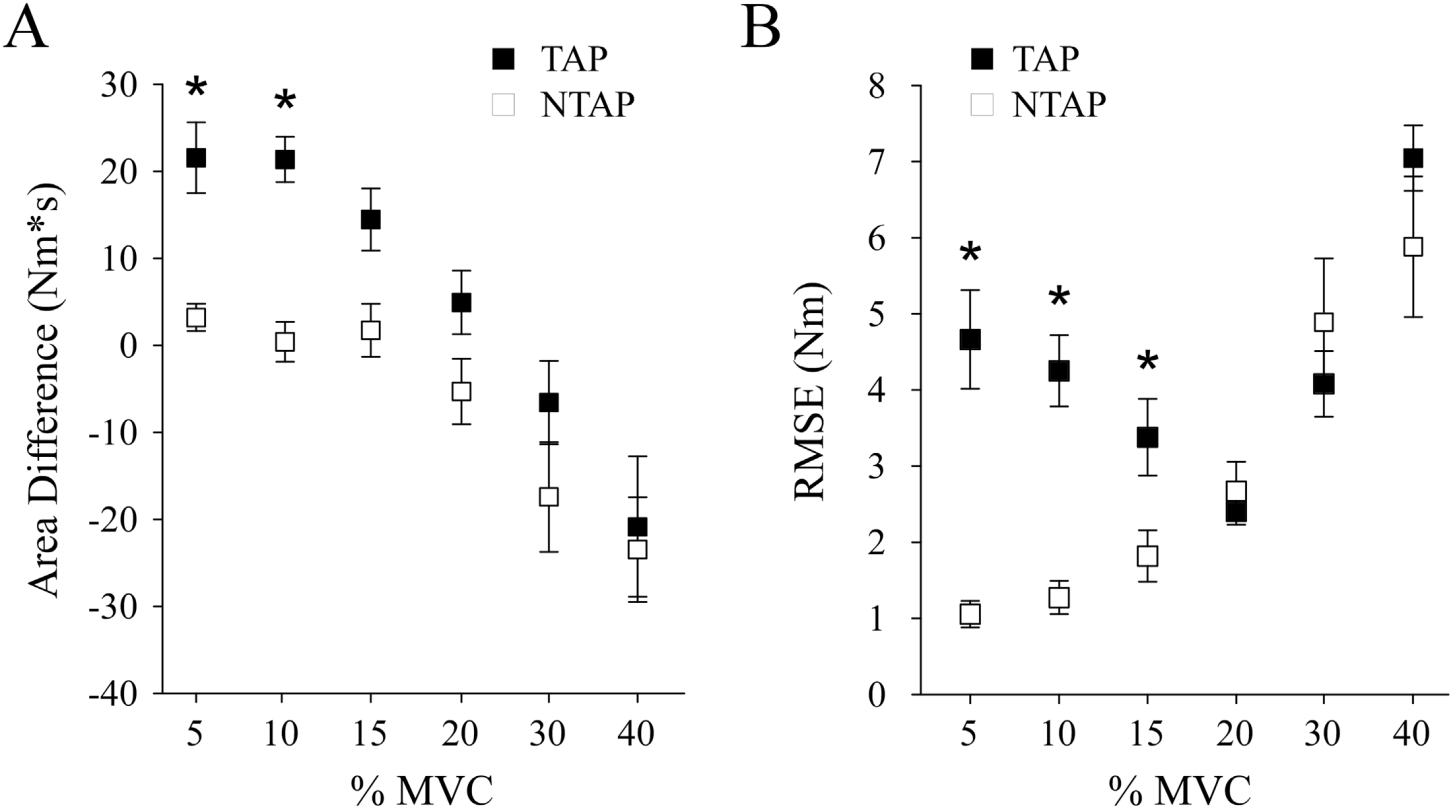
Torque output measures. Area (A) and RMSE (B) for each target torque level. Asterisks indicate significant differences between TAP and NTAP values (p < 0.05). Values are means ± SE.

To determine the effect of patellar tendon tapping on knee flexor motor output, we examined measures of torque magnitude, error and variability during the last 5 seconds of the knee flexion contraction (POST). Knee flexion torque magnitude was greater during the TAP condition compared with the NTAP condition (tendon tap condition main effect, p = 0.001; refer to Fig 3). We also observed an interaction between tendon tapping and target level (%MVC) on torque magnitude (Fig 4). Torque magnitude, relative to the target, was greater at lower target levels (5%, 10%, 15%) following tendon taps, while there was no difference in torque magnitude at higher target levels between TAP and NTAP conditions (20%, 30%, 40%; magnitude: tendon tap condition × effort interaction, p = 0.045; Fig 4A). Similarly, torque error was greater during the TAP condition compared with NTAP (RMSE: tendon tap condition main effect, p = 0.002). Errors were exacerbated after tendon taps at the lower target torque levels (RMSE: tendon tap condition × effort interaction, p < 0.001; Fig 4B). We found no statistical differences in torque variability between TAP and NTAP conditions (CV: mean ± stdev: 2.94% ± 0.35 vs. 2.75 ± 0.21; p = 0.291) or target level (5%: 3.14 ± 0.27%; 10%: 2.58 ± 0.25%; 15%: 2.51 ± 0.22%; 20%: 2.62 ± 0.33%; 30%: 2.99 ± 0.48%; 40%: 3.24 ± 0.37%; p = 0.169).

To determine the influence of Ia afferent feedback from the antagonist muscles (quadriceps) on the amplitude of agonist muscle (hamstrings) activity, we compared the RMS amplitude of the MH EMG between TAP and NTAP conditions. We observed a trend of greater percent change in MH EMG amplitudes during the TAP (%Δ MH: 3.57 ± 1.94%) compared with the NTAP condition (%Δ MH: -0.37 ± 1.81%), but these findings were not significantly different (tendon tap condition main effect: MH, p = 0.14). As expected, subjects exhibited greater changes in MH EMG amplitude with effort (target level main effect: %Δ MH, p < 0.001). Similar to the torque findings, subjects exhibited a larger percent change in MH EMG amplitude during the lower target levels compared with higher target levels (Post-hoc: %Δ MH, 10% > 30%, 40%, p < 0.05).

We quantified the P-P reflex amplitude to determine whether the reflex amplitude differed among the target levels. There was a significant main effect of target level (p = 0.003). We observed a decrease in the P-P reflex responses from the 5% (1.2 ± 0.84%) target level to the 40% (0.7 ± 0.19%) target level, but the post-hoc comparisons did not reveal any significant differences.

We also quantified a co-activation ratio between the RMS of the MH and averaged RMS of the quadriceps muscles during the POST contraction. The co-activation ratio was significantly (p = 0.042) lower for the TAP (3.1 ± 0.23%) compared with the NTAP (3.4 ± 0.29%) condition. We also observed significant differences in the co-activation ratios among the target levels (p = 0.001). Post-hoc comparisons showed the co-activation ratio for the 10% MVC trial was greater than the ratio from 15%, 20%, 30% and 40% (p < 0.05 for all comparisons).

### Effect of antagonist Ia sensory feedback on low frequency oscillations

**Fig 5:**
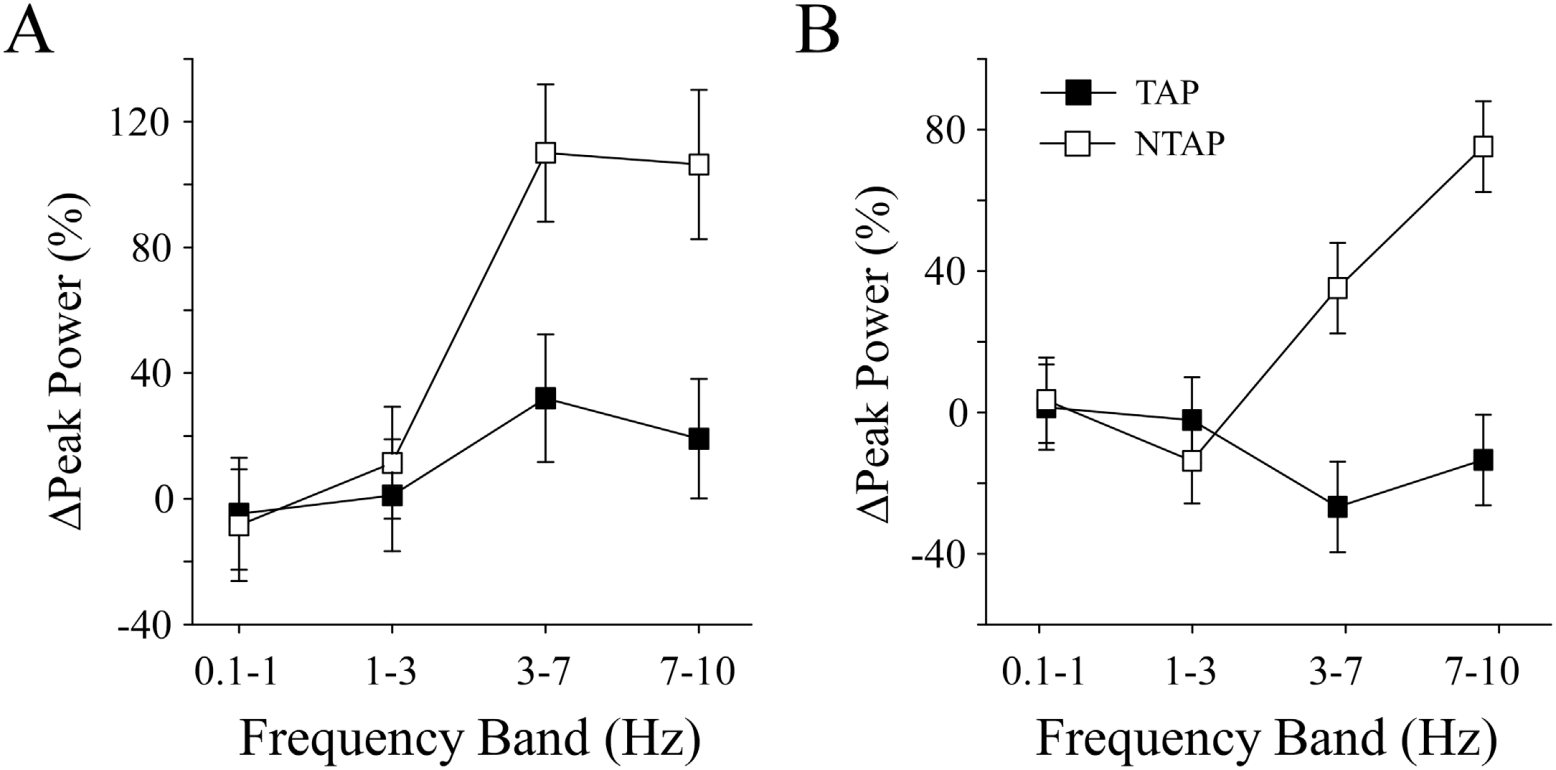
Torque Spectral Analysis. At lower torque target levels (10% MVC (A) and 15% MVC (B)), subjects exhibited smaller changes in peak power within 3–10 Hz following tendon tap perturbations. The data presented here are during the POST segments. Values are means ± SE.

We also examined how Ia feedback through tendon tapping of the antagonist muscles affected the power of low frequency oscillations (< 10 Hz) of knee flexion torque. Overall, the change in peak power within the torque output was *lower* (tap condition main effect, p = 0.001) during TAP trials (Δ% peak power, 6.19 ± 3.22%) compared with NTAP trials (Δ% peak power, 20.97 ± 3.31%). This difference was significantly lower (tap condition x effort x frequency band interaction, p = 0.01) at 10% and 15% MVC target torque levels (Fig 5).

### Hamstrings EMG power changes

We observed that tendon tapping affected the power within the MH EMG. We found a greater increase in peak power across all frequency bands (13–30, 30–60 and 60–100 Hz) following tendon tapping (TAP) compared to trials without tendon taps (NTAP) (Δ% MH peak power—tap condition main effect, p = 0.01).

## Discussion

The results from this study indicate that tendon taps of the antagonist muscle affect voluntary control of the agonist muscle differently at different levels of contraction. Tendon tap input (presumably Group Ia input) from the antagonist caused subjects to produce greater errors in torque output during lower contraction levels (5%, 10%, and 15% MVC). In contrast, during higher contraction levels (20%, 30%, and 40%), the error in torque output was not significantly different with and without patellar tendon taps. These findings suggest that during relatively low muscle activity there is a kinesthetic deficiency in the ability to correct motor output after sensory disturbances.

### Effects of antagonist tendon tapping on agonist motor output

One explanation for the increase in knee flexion torque error observed during lower contraction levels is that subjects perceived a change in quadriceps muscle length following the patellar tendon taps. Striking the patellar tendon elicits a stretch reflex of the quadriceps muscles, causing first a stretch and subsequently a muscle contraction. Because the task required subjects to maintain a constant knee flexion torque, the brief reflex-induced contraction of the knee extensor muscles may have been perceived as a change in net torque output. A mismatch between sensory feedback and the descending motor commands to the agonist muscle may cause subjects to perceive the sensory signal as an error and overcompensate for the disturbance to meet the demands of the task. Other studies have demonstrated similar behaviors in motor output when sensory feedback is altered. For example, during force matching tasks, altering stretch-related sensory feedback via tendon vibration causes an overestimation in force of the unperturbed arm [12].

In contrast, we did not observe differences in error at the higher contraction levels (20%, 30%, and 40% MVC) between trials with and without tendon tapping. Because there was no increase in agonist-antagonist co-activation with increasing torque levels it is unlikely that the reduced error is simply due to increased antagonist co-activation. Other studies have shown that co-contraction can influence torque output [31] and future studies will examine the interaction of co-contraction and Ia input. Alternatively, it is possible that at higher contraction levels, the sensory inputs received from patellar tendon tapping may be too small in comparison to the hamstrings activation. This may have reduced the effectiveness of the patellar tendon tap inputs to alter hamstrings activity.

These data likely suggest that a greater amount of descending input onto the motoneuron pool in combination with ascending input from the agonist muscles may have outweighed the sensory input from the antagonist. This would lessen conflicting sensory feedback errors perceived during the task. Changes in the gain of the Ia system can influence force regulation, in which decreased Ia input is associated with increased steadiness [32]. In the current study, the absolute magnitude of our Ia input was constant in each condition, and the descending input increased (as evidenced by increased MH activation), which likely resulted in a relative decrease in the contribution of quadriceps Ia input to the torque output of the hamstrings. Although it is possible that at higher loads there was decreased gamma drive [33] or increased Ia presynaptic inhibition [34], which would have decreased the relative efficacy of the antagonist input, or there is less sensory weighting given to the antagonist muscle at higher contractions levels compared to the lower contraction levels.

### Possible mechanisms

The increased error during lower contraction levels may be related to a cortical strategy to compensate for sensory disturbances. Although we do not have direct evidence for cortically-related changes in motor output following patellar tendon tapping, it has been suggested that changes in oscillations in motor output are related to changes in cortical drive to the motoneurons [35–37]. In the current study, we observed differences in the power spectrum of torque output within 3–10 Hz between TAP and NTAP conditions at low target levels (power spectrum is only for the POST segments of the contraction; Fig 5). During the NTAP condition, the change from visual feedback to no visual feedback may have caused subjects to rely less on cortical strategies to maintain the correct knee flexion output. In contrast, during the TAP condition there was little to no change in power within the 3–10 Hz band, which may suggest greater cortical involvement. Tracy et al. [8] observed similar oscillatory changes during a visuomotor isometric task. During the task, removal of visual feedback increased the power of knee extensor output within the 8–12 Hz frequency band, which suggests a change in control strategy involving greater involuntary processes [8]. Similarly, others have attributed common drive to differences in motor output between antagonist-agonist muscle activity [38,39]. Although, we did not see any large contribution from the antagonist muscle post tendon tapping, and this may depend on the joint position which our methods did not allow [38].

While spinal mechanisms may play a role in the responses, we do not think our findings are strongly related to spinal mechanisms. If the observed increase in error was due to simple summation of inputs at the level of the spinal cord, we would expect subjects to undershoot the target (produce less knee flexion) because the stretch reflex of the quadriceps muscle would theoretically inhibit the MN pool of the hamstrings.

### Functional implications

In healthy controls we showed that torque error is larger at lower versus higher loads in response to stimulation of antagonist Ia pathways. The overcompensation in knee flexion torque and error may ultimately be a stabilization strategy to prevent loss of balance or injury. In the current study, the motor goal is to produce a certain degree of knee flexion torque and the quadriceps reflex response is counterproductive to the goal of the motor command. The kinesthetic deficiency and error at lower loads may be accounted for through visual input in healthy controls. However, the deficiency at low loads may contribute to unbalanced activity in the hamstrings and quadriceps muscles, which is purported to contribute to knee ligament tears [40].

With respect to patient populations, patients with stroke have increased excitability of Ia pathways [3,4] and impairments in low-level force regulation [41–43] during isometric contractions. Although the mechanisms of impaired force regulation post stroke have not been fully explained, there is indirect evidence of altered coordination of agonist-antagonist activity and increased involvement of spinal pathways with and without visual feedback. We predict that the effects seen in the present study might be exaggerated in the stroke population and could potentially contribute to low-level force regulation deficits. Our findings may also have implications for recovery from sports related injuries that require exercises be performed with reduced weight bearing status, but in a functionally relevant task. Specifically, unloading the legs too much could be detrimental to control and there may be threshold levels of force needed for improving active range of motion without compromising balance responses. This is supported by other studies which have shown load-related effects on walking kinematics and kinetics in patient populations [44].

## Supporting Information

S1 Data (XLSX)
